# Supplementary material for: Evolution towards simplicity in bacterial small heat shock protein system
Source: eLife. 2023 Dec 8;12:RP89813. doi: 10.7554/eLife.89813 (PMC10708888; doi:10.7554/eLife.89813)
Supplement: Supplementary file 3. — For each position amino acids reconstructed with posterior probability higher than 0.2 are shown. Single-letter symbols of reconstructed amino acids are followed by posterior probability of reconstruction (in brackets). Positions at which most likely amino acid differ between AncA0 and AncA1 are marked in bold and italics. Posterior probabilities were estimated using the FastML program based on the Maximum Likelihood and Empirical Bayes method. [file elife-89813-supp3.docx]

**Supplementary file 3** **Posterior probability statistics for ancestral sequence reconstruction of AncA_0_ and AncA_1_ nodes:** For each position amino acids reconstructed with posterior probability higher than 0.2 are shown. Single – letter symbols of reconstructed amino acids are followed by posterior probability of reconstruction (in brackets). Positions at which most likely amino acid differ between AncA_0_ and AncA_1_ are marked in bold and italics. Posterior probabilities estimated using FastML program based on Maximum Likelihood and Empirical Bayes method.

| Position Number | Posterior probabilities | |
| --- | --- | --- |
|  | **AncA_0_** | **AncA_1_** |
| 1 | M (0.999926) | M (0.999866) |
| 2 | R (0.999613) | R (0.999411) |
| 3 | N (0.999721) | N (0.999542) |
| 4 | F (0.999497) | F (0.999873) |
| 5 | D (0.999313) | D (0.999001) |
| 6 | L (0.999064) | L (0.999692) |
| 7 | S (0.998966) | S (0.997905) |
| 8 | P (0.99996) | P (0.999935) |
| 9 | L (0.999823) | L (0.999728) |
| 10 | Y (0.999937) | Y (0.999902) |
| 11 | R (0.999613) | R (0.999411) |
| 12 | S (0.999127) | S (0.998633) |
| 13 | A (0.999325) | A (0.99838) |
| 14 | I (0.998482) | I (0.997912) |
| 15 | G (0.999955) | G (0.999926) |
| 16 | F (0.999931) | F (0.99989) |
| 17 | D (0.999313) | D (0.999001) |
| 18 | R (0.999613) | R (0.999411) |
| 19 | L (0.999823) | L (0.999728) |
| 20 | F (0.999754) | F (0.97628) |
| 21 | N (0.999721) | N (0.999542) |
| 22 | L (0.999812) | L (0.999727) |
| 23 | L (0.9998) | L (0.999727) |
| 24 | E (0.999305) | E (0.999025) |
| 25 | S (0.961991) | S (0.995727) |
| 26 | N (0.988107) | N (0.999069) |
| 27 | Q (0.999869) | Q (0.999782) |
| 28 | N (0.620376) S (0.354683) | N (0.671642) |
| 29 | Q (0.999869) | Q (0.999779) |
| 30 | S (0.999045) | S (0.998513) |
| 31 | N (0.999721) | N (0.999542) |
| 32 | G (0.999955) | G (0.999926) |
| 33 | G (0.999955) | G (0.999926) |
| 34 | Y (0.999937) | Y (0.999902) |
| 35 | P (0.99996) | P (0.999935) |
| 36 | P (0.99996) | P (0.999935) |
| 37 | Y (0.999937) | Y (0.999902) |
| 38 | N (0.999721) | N (0.999542) |
| 39 | V (0.998095) | V (0.997517) |
| 40 | E (0.999305) | E (0.999025) |
| 41 | L (0.999823) | L (0.999728) |
| 42 | V (0.998095) | V (0.997517) |
| 43 | D (0.999279) | D (0.998143) |
| 44 | E (0.999129) | E (0.999009) |
| 45 | N (0.999721) | N (0.999542) |
| 46 | H (0.999623) | H (0.999857) |
| 47 | Y (0.999937) | Y (0.999902) |
| 48 | R (0.999613) | R (0.999411) |
| 49 | I (0.998482) | I (0.997912) |
| 50 | ***A (0.986377)*** | ***T (0.983415)*** |
| 51 | I (0.998482) | I (0.997911) |
| 52 | A (0.999353) | A (0.999) |
| 53 | V (0.998095) | V (0.997517) |
| 54 | A (0.999353) | A (0.999) |
| 55 | G (0.999955) | G (0.999926) |
| 56 | F (0.999931) | F (0.99989) |
| 57 | A (0.999353) | A (0.998999) |
| 58 | ***E (0.993163)*** | ***Q (0.991222)*** |
| 59 | S (0.980818) | S (0.993494) |
| 60 | E (0.999305) | E (0.999025) |
| 61 | L (0.999823) | L (0.999728) |
| 62 | D (0.94017) | D (0.991033) |
| 63 | I (0.998482) | I (0.997912) |
| 64 | T (0.999463) | T (0.999145) |
| 65 | ***A (0.976453)*** | ***S (0.978346)*** |
| 66 | ***Q (0.703474) H (0.296138)*** | ***H (0.994492)*** |
| 67 | D (0.999258) | D (0.998997) |
| 68 | N (0.999721) | N (0.999542) |
| 69 | L (0.999261) | L (0.981563) |
| 70 | L (0.999823) | L (0.999728) |
| 71 | I (0.997518) | I (0.997483) |
| 72 | V (0.998094) | V (0.997516) |
| 73 | ***K (0.940376)*** | ***R (0.984241)*** |
| 74 | G (0.999955) | G (0.999926) |
| 75 | A (0.998624) | A (0.998938) |
| 76 | H (0.999878) | H (0.999865) |
| 77 | A (0.997391) | A (0.889298) |
| 78 | ***G (0.977685)*** | ***E (0.991155)*** |
| 79 | E (0.999217) | E (0.999017) |
| 80 | Q (0.99967) | Q (0.999775) |
| 81 | P (0.752893) | P (0.8025) |
| 82 | E (0.998753) | E (0.998985) |
| 83 | R (0.999607) | R (0.999292) |
| 84 | T (0.987703) | T (0.804431) |
| 85 | Y (0.999937) | Y (0.999902) |
| 86 | L (0.999823) | L (0.999728) |
| 87 | Y (0.999937) | Y (0.999902) |
| 88 | Q (0.999869) | Q (0.999782) |
| 89 | G (0.999955) | G (0.999926) |
| 90 | I (0.998482) | I (0.997912) |
| 91 | A (0.999353) | A (0.999) |
| 92 | E (0.999305) | E (0.999025) |
| 93 | R (0.999613) | R (0.999411) |
| 94 | N (0.999721) | N (0.999542) |
| 95 | F (0.999931) | F (0.99989) |
| 96 | E (0.999305) | E (0.999025) |
| 97 | R (0.999613) | R (0.999411) |
| 98 | K (0.999614) | K (0.99943) |
| 99 | F (0.999931) | F (0.99989) |
| 100 | Q (0.999869) | Q (0.999782) |
| 101 | L (0.999823) | L (0.999728) |
| 102 | A (0.999353) | A (0.999) |
| 103 | E (0.998666) | E (0.98408) |
| 104 | H (0.984462) | H (0.999627) |
| 105 | I (0.991805) | I (0.840342) |
| 106 | H (0.979793) | H (0.990368) |
| 107 | V (0.989116) | V (0.996634) |
| 108 | R (0.968539) | R (0.997839) |
| 109 | ***G (0.99667)*** | ***D (0.991238)*** |
| 110 | A (0.999353) | A (0.999) |
| 111 | ***N (0.99357)*** | ***R (0.988165)*** |
| 112 | L (0.999823) | L (0.999728) |
| 113 | E (0.987968) | E (0.998552) |
| 114 | N (0.999721) | N (0.999542) |
| 115 | G (0.999955) | G (0.999926) |
| 116 | L (0.999823) | L (0.999728) |
| 117 | L (0.999823) | L (0.999728) |
| 118 | Y (0.999936) | Y (0.999865) |
| 119 | I (0.998472) | I (0.997904) |
| 120 | D (0.999116) | D (0.998989) |
| 121 | L (0.999751) | L (0.999724) |
| 122 | E (0.999305) | E (0.999025) |
| 123 | R (0.999613) | R (0.999411) |
| 124 | V (0.770855) I (0.228497) | V (0.794577) I (0.204744) |
| 125 | V (0.904419) | V (0.990099) |
| 126 | P (0.99996) | P (0.999935) |
| 127 | E (0.999305) | E (0.999025) |
| 128 | A (0.988944) | A (0.985575) |
| 129 | M (0.999359) | M (0.999806) |
| 130 | K (0.99958) | K (0.999428) |
| 131 | P (0.99996) | P (0.999935) |
| 132 | R (0.999613) | R (0.999411) |
| 133 | R (0.999523) | R (0.999404) |
| 134 | I (0.998482) | I (0.997912) |
| 135 | E (0.999209) | E (0.997485) |
| 136 | I (0.998482) | I (0.997912) |
| 137 | ***N (0.511055) K (0.449598)*** | ***L (0.988567)*** |
| 138 | ***-*** | ***K (0.619166) S (0.322748)*** |
