# Supplementary material for: Evolution towards simplicity in bacterial small heat shock protein system
Source: eLife. 2023 Dec 8;12:RP89813. doi: 10.7554/eLife.89813 (PMC10708888; doi:10.7554/eLife.89813)
Supplement: Supplementary file 4. — (A) Branch model statistics for IbpA orthologs from Enterobacterales. Models assumed either branch between nodes AncA0 and AncA1 or entire Erwiniaceae clade as foreground; NS – Not significant. (B) Branch-site model statistics for IbpA orthologs from Enterobacterales. Models assumed either branch between nodes AncA0 and AncA1 or entire Erwiniaceae clade as foreground; NS – Not significant. [file elife-89813-supp4.docx]

**Supplementary file 4A Branch model statistics for IbpA orthologs from *Enterobacterales*:** Models assumed either branch between nodes AncA_0_ and AncA_1_ or entire *Erwiniaceae* clade as foreground; NS – Not significant

| **model** | **Np** | **lnL** | **ω estimates** | **LRT** | |
| --- | --- | --- | --- | --- | --- |
| **M0** | **154** | **-11631.91** | **ω=0.04** | **N/A** | |
| **Two – Ratio, A_0_-A_1_ branch as foreground** | **155** | **-11618.80** | **ω_0_=0.04** | **LRT = 26.22** | **P<0.001** |
|  |  |  | **ω_1_=999.00** |  |  |
| **Two – Ratio, Erwiniaceae clade as foreground** | **155** | **-11630.36** | **ω_0_=0.04** | **LRT = 3.11** | **NS** |
|  |  |  | **ω_1_=0.04** |  |  |

**Suplementary file 4B Branch - site model statistics for IbpA orthologs from *Enterobacterales*:** Models assumed either branch between nodes AncA_0_ and AncA_1_ or entire *Erwiniaceae* clade as foreground; NS – Not significant

| **model** | | **Np** | **lnL** | **site class** | **proportion** | **Background ω** | **foreground ω** | **LRT** | |
| --- | --- | --- | --- | --- | --- | --- | --- | --- | --- |
| **A_0_-A_1_ branch as foreground** | **A null** | **156** | **-11576.80** | **0** | **0.35** | **0.03** | **0.03** | **N/A** | |
|  |  |  |  | **1** | **0.01** | **1.00** | **1.00** |  |  |
|  |  |  |  | **2a** | **0.63** | **0.03** | **0.40** |  |  |
|  |  |  |  | **2b** | **0.01** | **1.00** | **0.40** |  |  |
|  | **A** | **157** | **-11565.20** | **0** | **0.88** | **0.03** | **0.03** | **LRT = 23.20** | **P<0,001** |
|  |  |  |  | **1** | **0.01** | **1.00** | **1.00** |  |  |
|  |  |  |  | **2a** | **0.10** | **0.03** | **999.00** |  |  |
|  |  |  |  | **2b** | **0.00** | **1.00** | **999.00** |  |  |
| ***Erwiniaceae* clade as foreground** | **A null** | **156** | **-11523.90** | **0** | **0.90** | **0.03** | **0.03** | **N/A** | |
|  |  |  |  | **1** | **0.03** | **1.00** | **1.00** |  |  |
|  |  |  |  | **2a** | **0.07** | **0.03** | **0.40** |  |  |
|  |  |  |  | **2b** | **0.00** | **1.00** | **0.40** |  |  |
|  | **A** | **157** | **-11570.20** | **0** | **0.98** | **0.03** | **0.03** | **LRT= - 92.51** | **NS** |
|  |  |  |  | **1** | **0.01** | **1.00** | **1.00** |  |  |
|  |  |  |  | **2a** | **0.01** | **0.03** | **1.00** |  |  |
|  |  |  |  | **2b** | **0.00** | **1.00** | **1.00** |  |  |
