# Supplementary material for: Evolution towards simplicity in bacterial small heat shock protein system
Source: eLife. 2023 Dec 8;12:RP89813. doi: 10.7554/eLife.89813 (PMC10708888; doi:10.7554/eLife.89813)
Supplement: Supplementary file 5. [file elife-89813-supp5.docx]

**Nucleotide sequences of *de novo* synthesized genes ordered from Genscript:**

***ancA0*:** ATGAGGAATTTTGATCTATCACCCTTATATAGAAGCGCGATCGGCTTTG

ATCGTCTGTTCAACCTGTTGGAGTCTAACCAGAATCAAAGCAACGGCGGTTATCCACCGTATAACGTTGAACTGGTGGATGAAAACCACTATCGTATCGCCATTGCTGTGGCGGGCTTCGCCGAGTCCGAATTGGACATCACCGCGCAGGACAATCTGCTTATCGTGAAAGGTGCGCATGCAGGTGAACAGCCGGAGCGCACGTACCTGTACCAAGGTATTGCGGAACGTAATTTTGAGCGCAAGTTCCAACTGGCGGAGCACATTCATGTTCGTGGTGCTAATTTGGAGAACGGCCTGCTCTACATCGACCTGGAGCGCGTCGTTCCGGAAGCAATGAAACCGCGTCGTATTGAAATCAACTAA

***ancA1***: ATGAGGAATTTTGATCTATCACCCTTATATAGAAGCGCAATTGGCTTTG

ATCGTCTGTTTAACTTGTTGGAGAGCAATCAAAACCAATCCAATGGCGGTTATCCACCGTATAACGTGGAATTGGTTGATGAAAACCACTACCGCATCACGATCGCCGTTGCGGGTTTCGCCCAGTCTGAACTGGACATCACCAGCCATGATAACCTGCTTATCGTTCGTGGTGCACATGCGGAGGAACAGCCGGAGCGCACCTATCTGTACCAGGGTATTGCGGAGCGTAATTTCGAACGTAAGTTCCAACTGGCGGAGCACATCCACGTGCGCGACGCTCGTCTGGAGAACGGCCTGCTCTACATCGACCTGGAGCGCGTCGTGCCGGAAGCTATGAAACCGCGTCGTATTGAAATTCTGAAATAA

***ancA0+7*:** ATGAGAAATTTTGATCTATCACCCTTATATAGGTCTGCGATCGGCTTCGATCGTTTGTTTAACTTGTTGGAGTCCAATCAAAACCAAAGCAATGGCGGTTATCCACCGTATAACGTGGAACTGGTGGACGAAAACCACTACCGCATCACCATTGCTGTGGCCGGTTTCGCCGAGAGCGAACTGGACATCACGGCGCATGATAATCTGTTAATTGTTCGTGGTGCGCACGCAGAGGAACAGCCGGAGCGCACCTATCTGTACCAGGGTATTGCTGAGCGCAACTTTGAACGCAAGTTCCAGCTGGCGGAGCACATCCATGTTCGTGATGCGCGTCTCGAGAACGGCCTGCTGTACATCGACCTGGAGCGTGTTGTCCCGGAAGCAATGAAACCGCGTCGTATTGAAATCCTGTAA

***ancA0 ACD:*** ATGGGAAGTTCACATCACCACCACCACCATTCTTCCGGCCTGGTCCCGCGTGGCAGCCATGGTGGTTATCCGCCATACAATGTTGAACTCGTGGATGAAAACCACTATCGTATTGCGATTGCTGTGGCGGGTTTTGCGGAGAGCGAACTGGACATCACCGCACAGGACAACCTGCTTATCGTGAAAGGTGCGCATGCTGGCGAACAACCGGAACGCACGTATCTGTACCAAGGTATTGCCGAGCGTAATTTCGAGCGCAAGTTCCAGCTGGCAGAGCACATCCACGTTCGTGGCGCGAACCTGGAGAACGGCTTGTTGTACATCGATCTGGAGAGATAA

***ancA0 Q66H G109D ACD:*** ATGGGATCTTCACATCATCACCACCACCACAGTTCCGGCCTGGTTCCGCGTGGTAGCCACGGCGGTTATCCACCGTATAACGTGGAATTGGTCGATGAGAATCATTATCGTATTGCGATCGCAGTTGCGGGTTTTGCCGAGAGCGAACTGGACATCACCGCACACGACAACCTGCTGATTGTTAAAGGTGCGCACGCTGGCGAACAGCCGGAACGTACGTACCTGTACCAAGGTATTGCGGAGCGCAACTTCGAGAGAAAGTTCCAGCTGGCGGAACATATCCACGTGCGTGATGCTAATCTCGAGAACGGCTTGTTATACATCGACCTGGAGCGCTAA

***his6 - sumo -C-peptide:*** ATGCATCATCATCATCATCACGGCAGCGGCCTGGTGCCGCGCGGCAGCGCTAGCATGTCGGACTCAGAAGTCAATCAAGAAGCTAAGCCAGAGGTCAAGCCAGAAGTCAAGCCTGAGACTCACATCAATTTAAAGGTGTCCGATGGATCTTCAGAGATCTTCTTCAAGATCAAAAAGACCACTCCTTTAAGAAGGCTGATGGAAGCGTTCGCTAAAAGACAGGGTAAGGAAATGGACTCCTTAAGATTCTTGTACGACGGTATTAGAATTCAAGCTGATCAGACCCCTGAAGATTTGGACATGGAGGATAACGATATTATTGAGGCTCACAGAGAACAGATTGGTGGTGGAGGGTCAGGTGGACCCGAAGCTATGAAACCGCGCCGTATTGAGATCAACTAA
